# From Data to Better Care: Consumer Wearables for Sleep and Circadian Rhythm Monitoring in Nursing Homes

**DOI:** 10.1093/geroni/igaf122.2063

**Published:** 2025-12-31

**Authors:** Kathy Richards, Brian Cox, Vanessa Aguilar, Robert Morgan, A Lynn Snow, Christine Hartmann

**Affiliations:** The University of Texas at Austin, Austin, Texas, United States; Alabama Research Institute on Aging, Tuscaloosa, Alabama, United States; The University of Alabama, Tuscaloosa, Alabama, United States; The University of Texas Health Science Center, Houston, Texas, United States; The University of Alabama, Tuscaloosa, Alabama, United States; University of Massachusetts Lowell, Lowell, Massachusetts, United States

## Abstract

The expanding availability of consumer-grade sleep tracking devices, like smartwatches, fitness bands, and smart rings, has been driven by advancements in wearable technology, miniaturized sensors, and artificial intelligence-driven analytics. This presentation reviews the state of the science for application of wearable technology to measure and monitor sleep and circadian rhythms in nursing home residents and describes how technologies have evolved over time. Key characteristics, including sensors, feedback to consumers, cloud service, raw data access, accuracy, cost, and capability will be discussed in light of the inherent opportunities and challenges for implementation of this technology in both nursing home research and practice. As these technologies become more sophisticated and widely available, they present new possibilities for integrating sleep and circadian rhythm tracking into both research and practice and may significantly improve residents’ health and well-being.

